# The Interaction between the DOCK7 Protein and the E2 Protein of  Classical Swine Fever Virus Is Not Involved with Viral Replication or Pathogenicity

**DOI:** 10.3390/v16010070

**Published:** 2023-12-30

**Authors:** Elizabeth Vuono, Elizabeth Ramirez-Medina, Ediane Silva, Keith Berggren, Ayushi Rai, Nallely Espinoza, Manuel V. Borca, Douglas P. Gladue

**Affiliations:** 1 Plum Island Animal Disease Center (PIADC), Agricultural Research Service, United States Department of Agriculture, Orient, NY 11957, USA; elizabeth.vuono@enkochem.com (E.V.); elizabeth.ramirez@usda.gov (E.R.-M.); ediane.silva@usda.gov (E.S.); keithab@princeton.edu (K.B.); ayushi.rai@usda.gov (A.R.); nallely.espinoza@usda.gov (N.E.); 2 National Bio-and Agro-Defense Facility (NBAF), Agricultural Research Service, United States Department of Agriculture, Manhattan, KS 66502, USA; 3 Oak Ridge Institute for Science and Education (ORISE), Oak Ridge, TN 37830, USA

**Keywords:** CSFV, classical swine fever, E2 glycoprotein, virus virulence, dedicator of cytokinesis, DOCK7

## Abstract

The classical swine fever virus (CSFV) particle consists of three glycoproteins, all of which have been shown to be important proteins involved in many virus functions, including interaction with several host proteins. One of these proteins, E2, has been shown to be directly involved with adsorption to the host cell and important for virus virulence. Using the yeast two-hybrid system, we have previously shown that CSFV E2 specifically interacts with the (DOCK7) dedicator of cytokinesis, a scaffolding protein. In this report, the interaction between E2 and DOCK7 was evaluated. To confirm the yeast two-hybrid results and to determine that DOCK7 interacts in swine cells with E2, we performed co-immunoprecipitation and proximity ligation assay (PLA). After demonstrating the protein interaction in swine cells, E2 amino acid residues Y65, V283, and T149 were determined to be critical for interaction with Dock7 by using a random mutated library of E2 and a reverse yeast two-hybrid approach. That disruption of these three residues with mutations Y65F, V283D, and T149A abrogated the Dock7-E2 protein interaction. These mutations were then introduced into a recombinant CSFV, E2DOCK7v, by a reverse genomics approach using the highly virulent CSFV Brescia isolate as a backbone. E2DOCKv was shown to have similar growth kinetics in swine primary macrophages and SK6 cell cultures to the parental Brescia strain. Similarly, E2DOCK7v demonstrated a similar level of virulence to the parental Brescia when inoculated in domestic pigs. Animals intranasally inoculated with 10^5^ TCID_50_ developed a lethal form of clinical disease with virological and hematological kinetics changes indistinguishable from that produced by the parental strain. Therefore, interaction between CSFV E2 and host DOCK7 is not critically involved in the process of virus replication and disease production.

## 1. Introduction

Devastating losses to the swine industry can result from outbreaks of classical swine fever (CSF). The CSF virus (CSFV) is highly contagious and often produces a lethal disease in swine. CSFV, like other pestiviruses, is an enveloped virus containing a positive-stranded RNA genome of approximately 12.5 kb, which is transcribed as a 3898-amino-acid polyprotein that is then cleaved to four structural and seven to eight nonstructural proteins [1]. The viral RNA is associated with the Core protein. Three glycoproteins (E^rns^RNS, E1, and E2), which are found in the viral envelope, are the structural proteins. In the last few years, studies have been carried out to determine viral protein roles in different cellular processes that could affect virus replication and virus virulence in the domestic pig, the natural host [2,3,4,5,6,7,8,9,10,11]. In the past several years, host proteins have been identified that are involved in protein–protein interactions with structural CSFV proteins. As examples, the Core protein was shown to specifically bind SUMO1 (small ubiquitin-related modifier 1), IQGAP1 (IQ motif containing GTPase activating protein 1), UBC9 (Ubiquitin Conjugating enzyme 9), and HB (Hemoglobin subunit beta) proteins [12,13,14,15]. Disrupting these protein interactions resulted in changes in virus replication and, in some cases, virus pathogenesis. The glycoprotein E^rns^ protein and the Laminin receptor protein play a critical role during cellular attachment [16]. The CSFV viroporin [17], p7, binds the calcium signal-modulating cyclophilin ligand (CALMG) [18], the microtubule-associated protein RP/EB family member 1 (MAPRE1), and the voltage-dependent anion channel 1 (VDAC1) [19], which all aid in the role of regulating viroporin function to regulate cellular homeostasis. Glycoprotein [19] E2 binds several host proteins during virus replication including actin [20], Anx2 (Annexin 2) [21], Trx2 (Thioredoxin) [22], MEK2 (mitogen-activated protein kinase 2) [23], PPP1CB (protein phosphatase 1 catalytic subunit beta) [24], SERTAD1 (SERTA domain containing protein 1) [25], CCDC115 (coiled coil domain containing 115) [26], ABCAM [19], and DCTN6 (dynactin subunit 6) [27]. The importance of each individual protein–protein interaction varies in terms of affecting virus replication in primary swine macrophages and in swine.

Using a yeast two-hybrid large-scale screening approach, we previously reported that the host dedicator of the cytokinesis 7 (DOCK7) protein and the CSFV E2 protein specifically interacts in yeast cells [28]. DOCK7 is a guanine nucleotide exchange g factor (GEF) that has shown GEF activity toward RAC1 and RAC3, two Rho Small GTPases. DOCK7 is a member of the Dock-C subfamily of the DOCK family, which has roles in activating G-proteins. Although the specific role for Dock7 in regards to virus infections has never been identified, other GEF proteins have been shown to play a role in virus infections [29] or in the mediation of the inflammatory response [30]. In this study, the protein–protein interaction between CSFV E2 and DOCK7 was confirmed in swine cells infected with CSFV by using two very different methodologies: co-immunoprecipitation, as a biochemical approach, and proximity ligation assay (PLA), which detects protein interactions intracellularly. In both cases, the E2-DOCK7 protein–protein interaction was successfully confirmed in a cell line of swine origin, SK6. To determine the potential function of this host–viral protein–protein interaction, the residues critical for mediating this interaction between E2 and DOCK7 were determined in E2. Based on this information and using a previously developed reverse genomics approach using an infectious DNA clone of the highly virulent Brescia isolate, a recombinant CSFV was made with mutations in specific amino acids in E2 that mediate this interaction. When tested in vitro in either SK6 cells or primary swine macrophages, a similar rate of replication was observed, and when tested in vivo in domestic swine, the level of virulence was similar to the parental virus. These results suggest that the interaction between E2 and DOCK7 when disrupted on its own is not critical for virus replication or pathogenesis.

## 2. Materials and Methods

### 2.1. Viruses and Cells

Swine kidney (SK6) cell cultures [5], negative for BVDV, were maintained in Dulbecco’s Minimal Essential Media (DMEM) (Gibco, Grand Island, NY, USA) containing 10% fetal calf serum (FCS) (Atlas Biologicals, Fort Collins, CO, USA). The parental CSFV is a virus derived from an infectious clone encoding the CSFV Brescia strain (BICv), which is used as a control virus in this study [5]. All titrations in this study of CSFV were performed using SK6 cell cultures as a substrate in 96-well plates (Costar, Cambridge, MA, USA). After 4 days in culture, virus was detected using a previously described immunoproxidase assay using a monoclonal antibody specific for E2, WH303 [3], and the Vectastain ABC kit (Vector Laboratories, Burlingame, CA, USA). Titers were calculated as described previously, expressed as TCID_50_/mL [31]. The limit of detection using this methodology is ≥1.8 TCID_50_/mL.

### 2.2. Immunoblotting and Antibodies

Immunoblotting was conducted with infected and mock-infected (control) samples. Cell cultures were washed once with ice-cold PBS and then lysed in RIPA buffer (Teknova, Hollister, CA, USA) in the presence of protease inhibitors (Roche, Basel, Switzerland). Cell extracts were run on NuPAGE 4–12% *w*/*v* Bis-Tris gels (Invitrogen, Carlsbad, CA, USA) and transferred to polyvinylidene difluoride (PVDF) membranes as described in the manufacturer instructions. Immunodetection of DOCK7 was performed using a polyclonal Anti-DOCK7 (catPA5-103779, Thermo Fisher Scientific, Waltham, MA, USA). Detection of CSFV E2 protein was performed using the monoclonal antibody WH303 [3]. As a secondary antibody, the Pierce Goat Anti-Mouse and Anti-Rabbit IgG peroxidase conjugated secondary antibody reagent (Cat #31430 and 31460, respectively, from Thermo Fisher Scientific, Waltham, MA, USA) was used. Western blots were imaged using an Azure C400 and analyzed with cSeries capture software version 1.6 (Azure Biosystems, Dublin, CA, USA).

### 2.3. Immunoprecipitation

Immunoprecipitation studies were carried out in triplicate using the Pierce Co-immunoprecipitation kit following manufacturer’s instructions (Thermo Fisher Scientific). Briefly, cell suspensions were washed with ice-cold PBS and lysed with the Pierce lysis buffer containing protease inhibitors (Roche, Basel, Switzerland). The anti-E2 WH303 [24] monoclonal antibody was conjugated to the Pierce beads and incubated with the cell lysates overnight. The beads were washed with Pierce Lysis buffer containing 0.1% Triton-X-100 (Sigma-Aldrich, St. Louis, MO, USA) and protease inhibitors (Roche) and eluted in Pierce elution buffer.

### 2.4. Proximity Ligation Assay

The Proximity Ligation Assay (PLA) was performed in triplicate using the Duolink-PLA kit (Sigma-Aldrich) following the manufacturer’s instructions. SK6 cells were seeded on 12 mm round coverslips (Thomas Scientific, Swedesboro, NJ, USA) using 24-well plates (Corning, Corning, NY, USA) at a concentration of 25,000 cells/well. Cells cultures were infected either with a high multiplicity of infection (MOI = 10), to allow the majority of the cells to become synchronically infected, or mock-infected (uninfected control cells) and, 24 h later, fixed with 4% formaldehyde *w*/*v* in PBS at room temperature for 20 min. Then, cells were treated with permeabilization buffer (0.3% Triton-X-100 in PBS) for 10 min. Fixed cells were then blocked with Duolink blocking buffer for 30 min at 37 °C. Cells were then incubated with the corresponding primary antibodies, anti-E2 WH303 and Anti-DOCK7 (cat# PA5-98661, Thermo Fisher Scientific, Waltham, MA, USA) at 4 °C for 1 h. Cells were then washed twice with Duolink (Sigma-Aldrich) wash buffer A and incubated with the PLUS and MINUS PLA probes for 1 h at 37 °C, followed by 2 washes with Duolink wash buffer A and a 30 min incubation at 37 °C with Duolink ligase in ligation buffer. Fixed cells were then washed twice with Duolink wash buffer A followed by incubation with Duolink polymerase in Amplification buffer at 37 °C for 100 min. The fixed cells were then washed twice with Duolink wash buffer B and mounted with Duolink PLA mounting medium with DAPI. A positive result is read by the observation of red spots using a Texas red filter.

### 2.5. Yeast Two-Hybrid Screening for Disruption of the E2-DOCK7 Reactivity

Plasmids harboring the E2 gene fused to the Gal4 binding domain (E2-BD), DOCK7 gene fused to the Gal4 activation domain (DOCK7-AD), were synthesized. Plasmids PGADT7 and HPRT1-AD were used as negative and positive controls in terms of interacting with CSFV E2, respectively, as previously described [26]. E2-BD was randomly mutated using a mutagenic PCR approach to give an average of 5 nucleotide substitutions across the E2 open reading frame. This random mutant library was constructed by Epoch Bioscience (Bothell, WA, USA), and screening was performed as previously described [25]. Sanger sequencing of the identified E2 mutants lacking DOCK7 binding often revealed that the mutated E2 protein contained stop codons or out-of-frame mutations, thus explaining why the loss of E2-DOCK7 interaction occurred. Plasmids that contained stop codons or out-of-frame mutations were discarded and not studied further. All E2 mutants were tested individually for their ability to bind DOCK7 and HPRT1 (hypoxanthine phosphoribosyl transferase 1), a previously identified [28] positive E2 protein interactor, or PGADT7 (negative control). This second selection was performed to discard any mutant E2 proteins that lost the ability to bind HPRT1 because of a gross structural change in E2.

### 2.6. Construction of CSFV E2ΔDOCK7 Mutant

A full-length infectious clone (IC) containing the complete genome of the virulent CSFV Brescia strain (pBIC) [5] was used as a template to incorporate the amino acid substitutions in the E2 gene to disrupt the E2-DOCK7 interaction. Residue substitutions Y65F V283D and T149A were introduced into the native E2 sequence to obtain the E2ΔDOCK7 construct. The E2ΔDOCK7 plasmid was obtained by DNA synthesis (Epoch Life Sciences, Sugar Land, TX, USA).

The IC harboring the E2ΔDOCK7 full-length genome was transcribed using the T7 MEGAscript system (Ambion, Austin, TX, USA). RNA was precipitated and electroporated into SK6 cells as previously described [14]. Recombinant virus E2ΔDOCK7 was then harvested and stocks were kept at −70 °C until use.

### 2.7. Animal Infection

The virulence of the E2ΔDOCK7 mutant was assessed using 30–40 lbs commercial breed swine (approximately 10 to 12 weeks old). Pigs were intranasally (IN) inoculated with 10^5^ TCID_50_ of either E2ΔDOCK7 or parental BIC virus (BICv). Presence of clinical signs associated with the disease (anorexia, depression, fever, purple skin discoloration, staggering gait, diarrhea, and cough) as well as body temperature were daily recorded throughout the experiment. Blood samples, obtained from the anterior vena cava in EDTA-containing tubes (Vacutainer), were collected at different times post-challenge (as shown in the corresponding figures). Total and differential white blood cell and platelet counts were obtained using a Beckman Coulter ACT (Beckman, Coulter, CA, USA). Animal experiments were performed under biosafety level 3 conditions in the animal facilities at Plum Island Animal Disease Center, following a strict protocol approved by the Institutional Animal Care and Use Committee (number 171.12-21-R, approved 12-09-21).

## 3. Results and Discussion

### 3.1. CSFV E2 and DOCK7 Interaction in CSFV-Infected Cells

**Figure 1:**
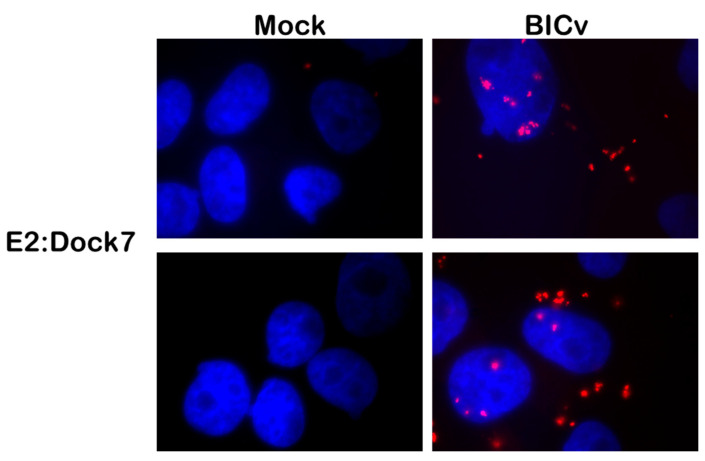
PLA for E2 and DOCK7 in SK6 cells infected or mock-infected with CSFV for 24 h with CSFV BICv (MOI = 10). Top and bottom panels on the right show different images with identical treatments. Magnification, ×1000. The figure shows results that are representative from one experiment performed three times, with a minimum of 100 cells analyzed in each experiment.

The interaction between CSFV E2 and DOCK7 was first identified in a large scale yeast two-hybrid screen using E2 as bait for host proteins; therefore, it was necessary to determine if the E2-DOCK7 interaction would occur in cells from the natural host of CSFV, swine cells. Two different approaches were used for this purpose, the first being a proximity ligation assay (PLA) [32] to detect if two proteins were in very close proximity within cell cultures, and the second being a biochemical approach using co-immunoprecipitation to detect if two proteins were specific binding partners. PLA, which will allow the detection of transient protein–protein interactions, was tested with a multiplicity of infection (MOI) of 10 with BICv to infect SK6 cells. Samples were harvested at 24 h post-infection (hpi). The results of the PLA confirmed that E2 and DOCK7 proteins interact in swine cells that are infected with CSFV, appearing as a distinct punctate location throughout the cell cytoplasm of infected cells, and it is virtually absent in the mock-infected cell preparations (Figure 1), indicating that in CSFV-infected cells, E2 specifically interacts with DOCK7, confirming the initial discovery as determined in the yeast two-hybrid large-scale screen to determine host binding partners for E2 protein.

**Figure 2:**
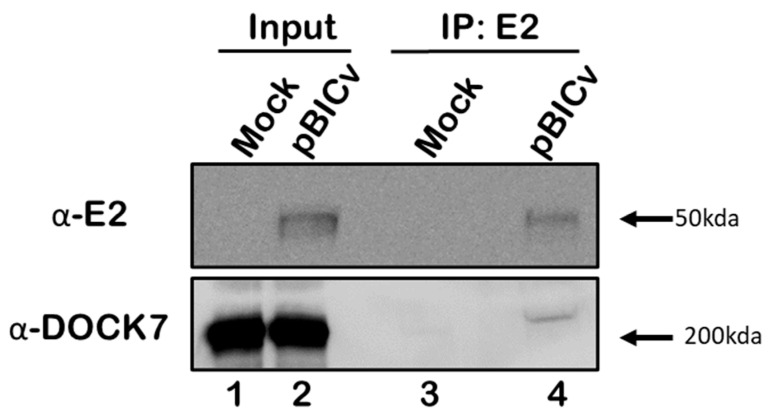
Co-immunoprecipitation studies between E2 and DOCK7 in CSFV- or mock-infected SK6 cells. Immunoprecipitation was performed using antibodies for E2. Western blots were performed with the indicated protein extracts for E2 and DOCK7, as indicated in Materials and Methods, with bands observed at the expected molecular weights, as indicated by the arrows of 50 kDa for E2 and 200 kDa for Dock7.

To further determine if the results from the PLA were the product of transient protein interactions or if E2 and DOCK7 were strongly bounded to each other, co-immunoprecipitation studies were conducted using cellular extracts of SK6 cells that were infected with CSFV. First, SK6 cells were infected with CSFV strain BICv at an MOI of 10 with cells being harvested 24 hpi, further described in Materials and Methods. Both mock infected and infected cell lysates were collected and were immunoprecipitated with WH303, an anti-E2 monoclonal antibody, and by Western blot detection for the presence or absence of DOCK7. A single band was observed showing a molecular mass of approximately 200 kDa, corresponding to the expected size of DOCK7 (Figure 2), clearly demonstrating that co-immunoprecipation occurred in CSFV-infected cell cultures between the structural protein E2 and host protein DOCK7. It should be noted that the DOCK7 that co-immunoprecipated with E2 was a slightly larger band that is observed in both the input cell lysate and pull down. Nonetheless, E2 and DOCK7 proteins’ interaction in CSFV-infected cells was demonstrated using two different, but specific, independent methodologies.

### 3.2. Identification of CSFV E2 Residues Critical for DOCK7 Interaction

Recombinant CSFV-containing amino acid substitutions that result in the disruption of the interactions between a virus protein and its host cell ligand are valuable instruments in examining the potential role of specific interactions between E2 and host proteins, as have been reported in other studies [24,25,26,27,33]. Earlier, we presented experiments, using alanine scanning mutagenesis, describing that the interaction between E2 and DOCK7 was a non-linear interaction and appeared to be conformation-dependent since the linear alanine scanning mutagenesis mutations failed to map E2 residues critical for mediating protein–protein interactions involving E2 and Dock7 [28]. In other viral proteins, alanine scanning mutagenesis successfully mapped amino acid residues in virus proteins shown to interact with host protein ligands [18]. Using a reverse yeast two-hybrid approach, amino acid residues of CSFV E2 were randomly mutated with an average of 5 mutations per E2 coding region; this library of random mutations identified the specific residues involved in the protein interaction with host DOCK7 and E2 by identifying mutants of E2 that lost the binding activity for DOCK7, as was described in a previous study [26]. To avoid mutations that introduce stop or frameshift mutations (which would produce false positive results), we checked the ability for these mutated forms of E2 to still interact with HPRT1, which specifically interacts with E2, ruling out the possibility of non-specific loss of protein binding due to conformational or folding changes. Constraining to these experimental conditions, there was only one E2 with point mutations that still lost the ability to bind to the DOCK7 protein, and we identified residue substitutions at positions Y65F, V283D, and T149A as being important for DOCK7 binding to E2 (Table 1). These residues were further used to analyze the process of CSFV replication and virulence in the absence of the DOCK7-E2 protein–protein interaction occurring.

### 3.3. Replication of the E2ΔDOCK7 Mutant in Cell Cultures

**Figure 3:**
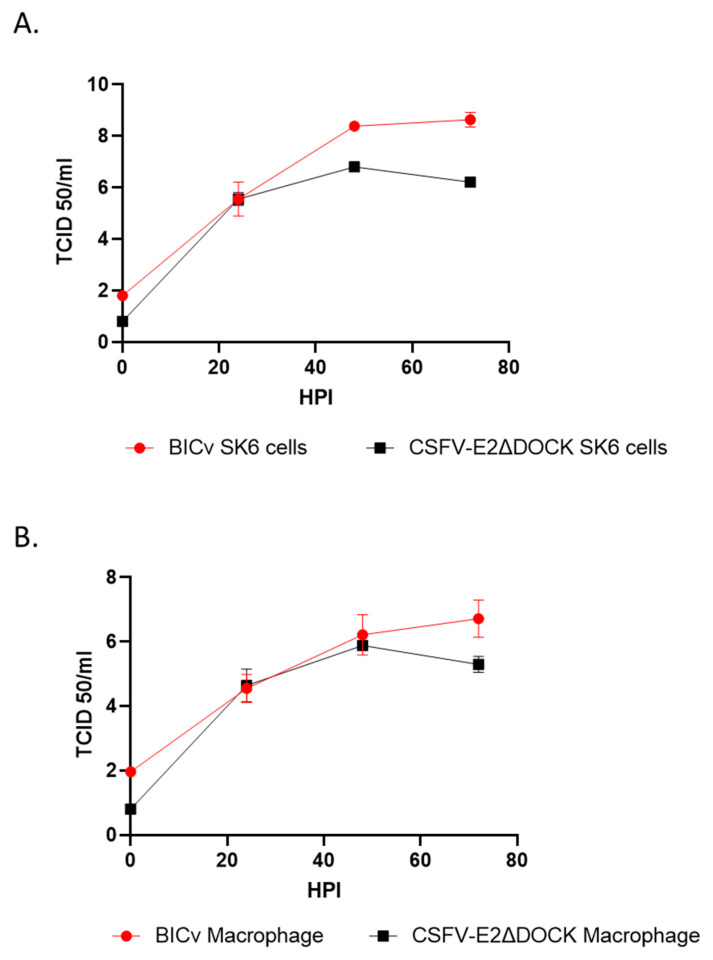
Growth kinetics in cells of swine origin. SK6 cells (**A**) and primary macrophage cell cultures (**B**) for E2ΔDOCK7v and parental BICv (MOI = 0.01). Two independent experiments were used to collect samples, at the indicated time points, and titrated in the corresponding cell substrate. Data represent means and standard deviations of three replicas. Sensitivity using this methodology for detecting virus is ≥log10 1.8 HAD_50_/mL.

Based on the results obtained by the reverse yeast two-hybrid experiments, a CSFV recombinant virus that harbored the same mutated residues in E2 containing amino acid changes, Y65F V283D and T149A, was developed, resulting in the recombinant virus E2ΔDOCK7v by a reverse genetic system using an infectious clone of BICv. The growth characteristics of the mutant virus E2ΔDOCK7v and those of parental BICv (the pBIC-derived virus) were compared in a multiple-step growth curve in swine cells using both SK6 and primary macrophage cultures (Figure 3). Using an MOI of 0.01 TCID_50_ per cell, both cell types were infected or mock-infected, and samples were collected every 24 h until 72 h post-infection (hpi). BICv displayed a higher level of replication in SK6 cells, reaching virus yields almost 2-fold greater than E2ΔDOCK7v. In primary swine macrophages, this growth reduction was slightly reduced, with only about a 1-fold decrease at 72 hpi in growth for E2ΔDOCK7v.

### 3.4. Assessment of E2∆DOCK7 Virulence in Swine

**Figure 4:**
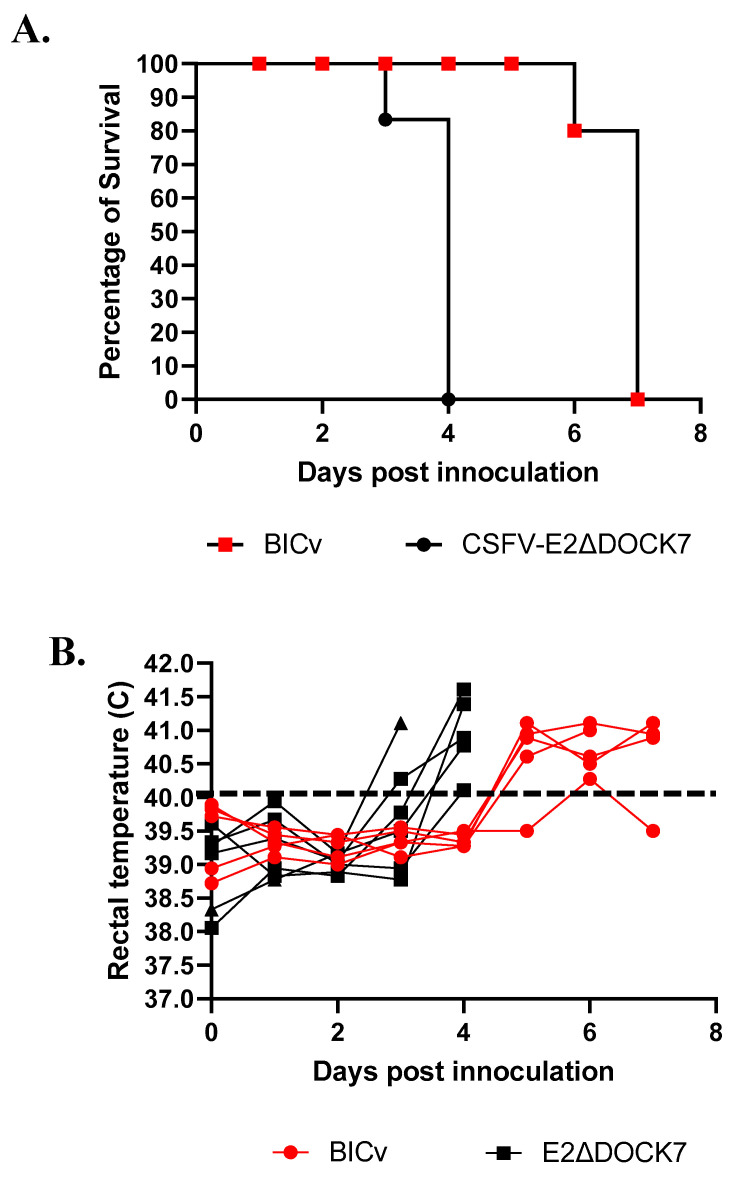
Evolution of lethality (**A**) and body temperature (**B**) in animals (5 animals/group) IN infected with 10^5^ HAD_50_ of either E2ΔDOCK7v or parental BICv. After analysis by using the Log-rank (Mantel–Cox) test and also the Gehan–Breslow–Wilcoxon test, it was observed that there was no significant statistical differences between the different groups of animals regarding their temperatures or lethality profiles.

To assess the possible role of changing the amino acid in glycoprotein E2 in E2ΔDOCK7v during virus replication and in the progression of disease in the natural host, domestic pigs were infected with E2ΔDOCK7v under experimental conditions. Two groups, composed by five animals weighing 30–40 lbs, were IN infected with 10^5^ TCID_50_ of either E2ΔDOCK7v or the parental BICv. When comparing these two groups on a daily basis, clinical signs associated with CSF were monitored. As expected, in the virulent BICv group, all the animals showed an increase in body temperature over 40 °C and displayed disease onset around day 5 pi with a quick evolution of the disease, with all animals euthanized by day 6–7 pi (Figure 4).

The group of animals infected with E2ΔDOCK7v showed similar signs as the BICv group, where the appearance of the clinical signs of the disease occurred slightly earlier than animals inoculated with BICv. The disease was first detected between days 3 and 4 pi, and later evolved to more severe disease, with all animals euthanized on day 4–5 pi. Therefore, the introduced amino acid substitutions into the E2 glycoprotein of E2ΔDOCK7v did not change the virulence of this virus in domestic pigs.

**Figure 5:**
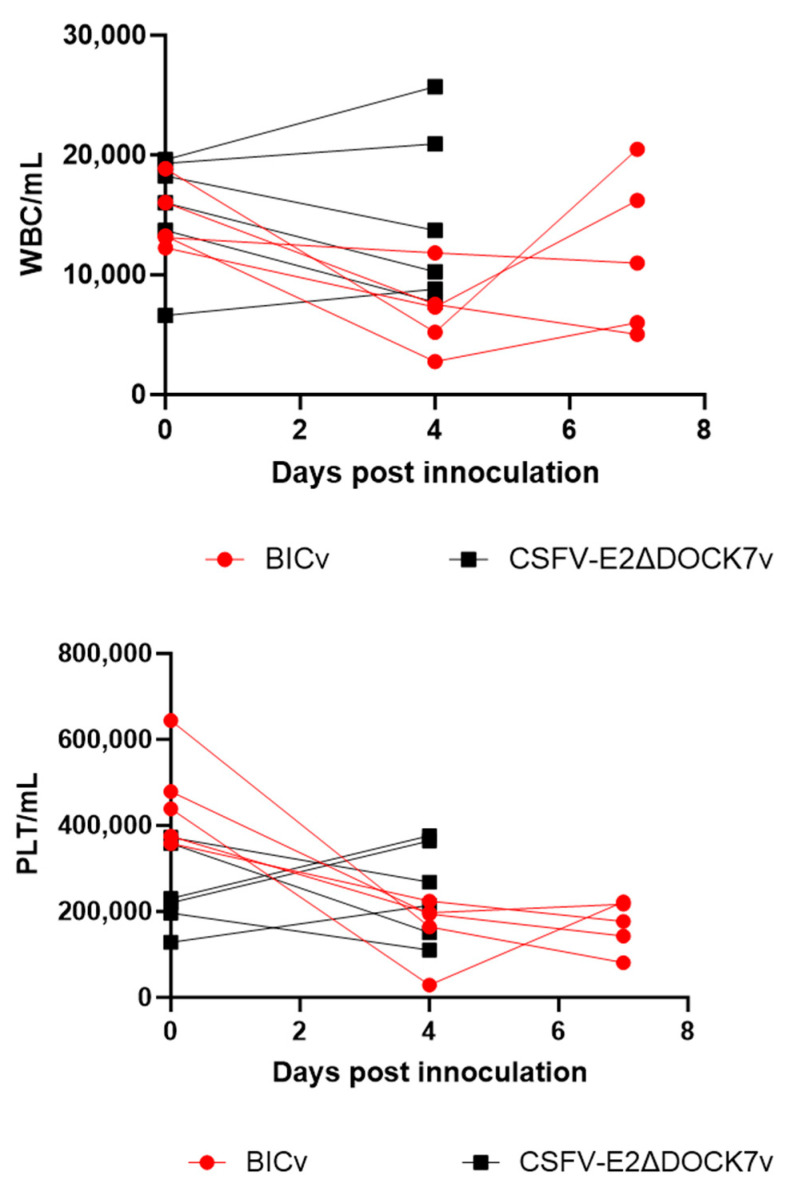
Hematological changes in pigs that were IN inoculated with 10^5^ TCID_50_ of E2ΔDOCK7v or BICv. Each curve represents individual animal values expressing concentration of platelets (PLTs) or white blood cells (WBCs)/mL of blood.

Hematological values followed the same evolution as did clinical disease for BICv, with both white blood cell and platelet counts dropping drastically by day 4 pi in animals inoculated with BICv, without further significant changes until death. However, there were different observations in E2ΔDOCK7v, where both the white blood cell and platelet counts increased by day 4 pi (Figure 5).

**Figure 6:**
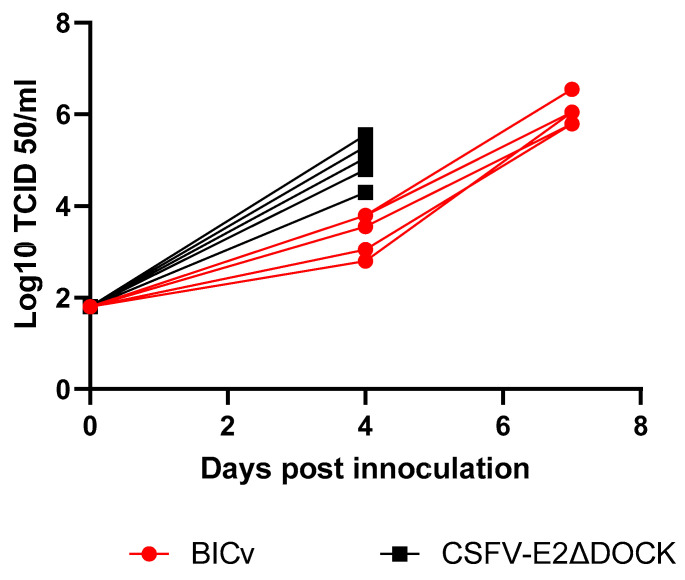
Viremia titers detected in pigs IN inoculated with 10^5^ TCID_50_ of either E2ΔDOCK7v or BICv. Each bar represents the average and standard deviation of the animal in each group at the corresponding time point.

The ability of recombinant E2ΔDOCK7v to replicate and cause disease in pigs was evaluated and a comparison between the viremia titers and those observed in the BICv-inoculated animals (Figure 6). Animals that were infected with BICv had previremia titers ranging from 10^2.2–3.6^ TCID_50_/mL by day 4 pi. Viremia values increased 10^5.2–6.2^ TCID_50_/mL by the day they were euthanized. The animals inoculated with E2ΔDOCK7v presented higher viremia titers by day 4 pi, ranging from 10^4–5.8^ TCID_50_/mL by day 4 pi, and were euthanized due to the severity of the clinical signs. Therefore, E2ΔDOCK7v does not present a virulent phenotype that has differences from BICv, the parental virulent virus. To confirm that the mutations in E2 were stable, the genome of the E2ΔDOCK7v obtained from infected pigs was sequenced. Results demonstrated that the mutation introduced in E2 remained stable.

The role of protein–protein interactions between the virus and host is critical for both virus replication and production of disease, as well as other critical processes. For CSFV, very little is known about virus–host protein–protein interactions in domestic pigs. Although studies on the identification and characterization of those interactions have increased in recent years, it is not clear the importance of those interactions regarding the molecular mechanisms developed by the virus for increasing virus replication and/or to manipulating the host immune response. We have previously reported the specific protein–protein interaction of several host and CSFV proteins. For instance, we identified and characterized the interaction of the structural CSFV Core protein with host proteins SUMO1, IQGAP1, UBC9, and OS9 [12,13,14]. In addition, we demonstrated the interaction of non-structural viroporin p7 with host CAMLG [18], as well as the interaction of the major structural glycoprotein E2 with host proteins ACADM [34], PPP1CB [24], DCTN6 [27], SERTAD1 [25], and CCDC115 [26]. These interactions between virus and host proteins commonly affect virus replication and, importantly, in some of these cases, may have an important role in the production of disease in pigs [12,13,18]. In addition, other laboratories have demonstrated that structural protein E2 interacts with several host proteins, such as cellular actin [20], Anx2 [21], Trx2 [22], and MEK2 [23]. Therefore, identifying and understanding the involvement of these virus–host interactions is critical to increasing the understanding of the molecular mechanisms involved during viral infection of the natural host.

In this study, we identified the specific amino acids in the CSFV E2 protein that are responsible for binding the host protein DOCK7, a guanine nucleotide exchange factor that plays a role in axon formation and neuronal polarization.

In this study, we characterize, for the first time, the cellular protein DOCK7 as an interaction partner for CSFV protein E2 in the infected cell. These results indicate that the E2-DOCK7 protein–protein interaction is not critically important for CSFV replication in vitro. Mutations that disrupt the E2-DOCK7 protein–protein interaction in YTH do not provoke significant changes in the E2ΔDOCK7v phenotype affecting virus replication in vitro and virus virulence during CSFV infection in the natural host, the domestic swine.

Gaining a better understanding of the importance of specific amino acid residues of CSFV proteins in binding cellular proteins allows us to gain a better understanding of how CSFV modulates the host to accomplish important aspects of virus replication or virulence, which is a key tool in the development of potential novel methods to block or decrease virus infection in the natural host. A better understanding of the host factors interacting with virus proteins at the amino acid level is a significant step towards better understanding the impact of potential mutations that occur between different emerging strains of CSFV, potentially helping to explain the differences observed with regards to viral virulence and pathogenesis.

## Figures and Tables

**Table 1:** CSFV protein E2 or E2-∆DOCK7 (Y65F V283D and T149A) protein–protein interactions with DOCK7, as identified using the yeast two-hybrid approach, where E2 or Lam as a negative control was coupled to the Gal4 binding domain with swine proteins DOCK7 or HPRT coupled to the Gal4 activation domain.

		Gal4 Binding Domain		
		Lam	E2	ΔDOCK7
**Gal4 Activation Domain**	T-ag	−	−	−
	DOCK7	−	+	−
	HPRT	−	+	+

## Data Availability

All Data is provided in this manuscript.
